# Spontaneous Tumor Lysis Syndrome in a Thoracic Burkitt Lymphoma: A Case Report

**DOI:** 10.7759/cureus.73084

**Published:** 2024-11-05

**Authors:** Alice Figueiredo, João Pimentel, Mafalda Figueira, Anabela Neves

**Affiliations:** 1 Oncology, Instituto Português de Oncologia de Lisboa Francisco Gentil, Lisboa, PRT; 2 Internal Medicine, Centro Hospitalar de Setúbal, Hospital de São Bernardo, Setúbal, PRT; 3 Anatomic Pathology, Centro Hospitalar Universitário de Lisboa Central, Lisbon, PRT; 4 Hematology, Centro Hospitalar de Setúbal, Hospital de São Bernardo, Setúbal, PRT

**Keywords:** acute spontaneous tumor lysis syndrome, burkitt lymphoma, lactic acidosis, mediastinal neoplasms, multiple organ failure, obstrutive shock, septic shock, thoracic burkitt lymphoma, tumor lysis syndrome

## Abstract

Burkitt lymphoma has a high proliferation rate and a significant risk of tumor lysis syndrome. Risk stratification and early identification are imperative since it is an oncological emergency.

We report the case of a 20-year-old woman, without relevant past medical history, admitted to the Emergency Department with a three-week history of fatigue, chest discomfort, productive cough, night sweats, myalgia, odynophagia, and holocranial headache. Laboratory findings included lactic acidosis, elevation of inflammatory markers, and high D-dimer levels. Computed tomography angiography identified a large mediastinal mass with critical compression of the right pulmonary artery. Subsequently, the patient developed spontaneous tumor lysis syndrome with hemodynamic and metabolic instability. Biopsy of the lesion revealed a Burkitt lymphoma with a ki67 of 100%, an unexpected diagnosis since sporadic Burkitt Lymphoma is atypical in mediastinal location.

Despite intensive hydration, rasburicase, and dexamethasone, progression to cardiogenic and obstructive shock required multiorgan support in the intensive care unit. After initial hemodynamic stabilization, targeted chemotherapy was initiated, but the patient’s condition further worsened, followed by bone marrow aplasia, refractory shock, and death.

The challenge of recognizing a serious illness in an apparently healthy young patient is highlighted, as well as maintaining a high level of suspicion of tumor lysis syndrome even in atypical circumstances.

## Introduction

Burkitt lymphoma (BL), an aggressive non-Hodgkin's lymphoma (NHL), is defined by the translocation of the C-myc gene on chromosome 8. There are three known clinical forms: endemic, sporadic, and immunodeficiency-associated. Endemic form occurs mainly on the African continent, typically presents as a facial tumor in children, and is associated with Epstein-Barr virus infection. On the opposite, sporadic form prevails in the North Atlantic regions and predominantly affects the abdominal cavity [1]. BL is extremely rare in adults, accounting for less than 2% of all adult lymphomas in Europe, and has a male prevalence [1,2].

One of its distinguishing features is an exceptionally fast growth, capable of doubling size within 24 hours [3]. This fast-growing behavior carries a high risk of tumor lysis syndrome (TLS), a clinical syndrome resulting from the massive destruction of tumor cells with the release of intracellular components into the systemic circulation, resulting in hyperphosphatemia, hyperkalemia, hyperuricemia, and hypocalcemia [4]. The most worrisome manifestations include renal failure, malignant arrhythmias, and seizures [5]. 

TLS is predominantly observed in hematologic malignancies, such as BL and acute leukemia, typically following the initiation of anticancer therapy, though, in rare cases, it can be triggered spontaneously in rapidly dividing neoplasms or high-burden tumors [4,5]. Although there is great heterogeneity in epidemiological data on TLS because incidence varies according to age, comorbidities, therapeutic sensitivity, diagnostic criteria used, and preventive measures applied [6,7], it has been reported in 6.1% of NHL patients [8], where the majority of cases are observed [9]. However, spontaneous TLS occurs in only 1.08% of hematological malignancies [10]. TLS is considered an oncological emergency due to high morbidity and mortality, with in-hospital reported mortality rates of 21% [9] and 32%, rising to 51% in the presence of renal complications, often requiring invasive organ support and intensive care unit (ICU) admission [11].

The Cairo and Bishop classification system currently defines TLS by distinguishing between laboratory and clinical syndrome. Laboratory TLS requires the concurrent development of at least two metabolic abnormalities during the same 24-hour period: uric acid>8.0 mg/dL, phosphorus>4.5 mg/dL, or potassium>6.0 mmol/liter (or a 25% increase from baseline), and corrected calcium<7.0 mg/dL or ionized calcium<1.12 mmol/liter (or 25% decrease from baseline). Clinical syndrome is defined by the presence of laboratory TLS and elevated serum creatinine levels, seizures, cardiac dysrhythmia, or death [7].

Once established, TLS is difficult to reverse, with severe complications in more than 60% of patients [9]; therefore, it is recommended to initiate prophylactic measures in high-risk individuals. According to a risk stratification model published by Cairo et al. and developed by an international expert panel in 2008, patients can be divided into high, intermediate, and low-risk groups after histological diagnosis. The diagnosis of BL is enough to place the patient in the high-risk category, requiring immediate prophylaxis with hydration and hypouricemic drugs [12]. Established TLS requires prompt similar measures with aggressive hydration plus allopurinol or rasburicase and organ support as per need [13].

## Case presentation

**Figure 1:**
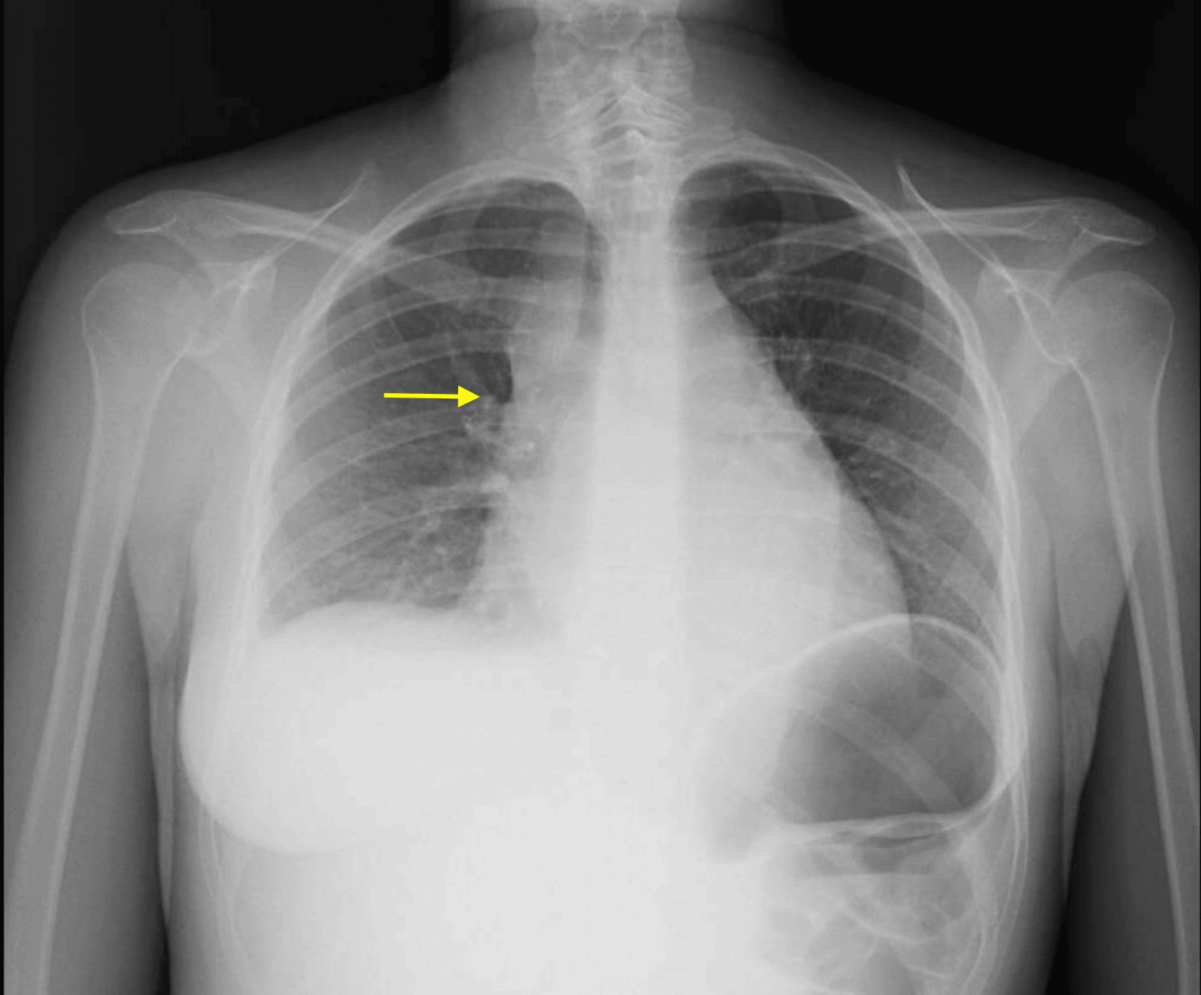
Chest X-ray performed at admission Enlargement of the superior and middle mediastinum is observed, with an increase in the projection of the superior vena cava (yellow arrow) and an increase in the projection of the hila, particularly on the right. Right pleural effusion and mild cardiomegaly were also noted.

We present the case of a 20-year-old patient without relevant past medical history and no history of chronic medication. The patient presented to the emergency department with a three-week history of fatigue, chest discomfort, productive cough, night sweats, myalgia, odynophagia, and holocranial headache. Although she had previously been diagnosed with community-acquired pneumonia and treated with empirical antibiotic therapy, she described lack of symptomatic relief. Due to her visible fatigue and reported chest pain, a blood gas analysis was performed, revealing lactic acidosis (Table 1), and blood tests revealed mild leukocytosis with neutrophilia and elevated C-reactive protein (Table 2). Due to a previous diagnosis of pneumonia with persistent productive cough and chest discomfort, a chest X-ray was performed, denoting widening of mediastinal shadow (Figure 1). Also, besides a low risk of pulmonary thromboembolism according to the Wells score, the high D-dimer levels (2,571 ng /mL) did not allow to safely exclude this diagnosis [14].

**Table 1:** Blood gas analysis at admission in the Emergency Department

Test	Measured	Reference Value
pH	7.45	7.35–7.45
pCO2	26 mmHg	35–48 mmHg
pO2	100 mmHg	83–108 mmHg
HCO_3_^-^	18.1 mmol/L	21.0–28.0 mmol/L
Na^+^	133 mmol/L	136–145 mmol/L
K^+^	3.8 mmol/L	3.4–4.5 mmol/L
Ca^2+^	1.14 mmol/L	1.15–1.27 mmol/L
Glucose	113 mg/dL	70–100 mg/dL
Lactate	3.1 mmol/L	<1.3 mmol/L

**Table 2:** Analysis at admission in the Emergency Department

Test	Results	Reference Value
Hemoglobin	11.70 g/dL	11.5–15 g/dL
Hematocrit	34.3%	36–46%
Globular median value	86.1 fL	82.1–97.7 fL
Corpuscular median hemoglobin	29.4 pg	27–32 pg
Leucocytes	13100 uL	4,500–11,400 uL
Neutrocytes	79.4%	41–75%
Monocytes	11.1%	2–11%
Eosinocytes	0.0%	0.4–6.0%
Basocytes	0.13%	<1.8%
Platelets	315,000 uL	150,000–350,000 uL
Prothrombin time	16.5 sec	9.4–12.5 sec
International normalized ratio	1.4	0.80–1.20
D-Dimers	2571 ng/mL	<500 ng/mL
Activated partial thromboplastin time	31.5 sec	25.1–36.5 sec
Glucose	95 mg/dL	70–105 mg/dL
Urea	19 mg/dL	15–40 mg/dL
Creatine	0.7 mg/dL	0.6–1.1 mg/dL
Sodium	140 mEq/L	136–146 mEq/L
Potassium	4.4 mEq/L	3.5–5.1 mEq/L
Chloride	102 mEq/L	98–107 mEq/L
Alanine aminotransferase	16 U/L	<55 U/L
Aspartate aminotransferase	12 U/L	5–34 U/L
Gamma-glutamyl transferase	20 U/L	9–36 U/L
Lactate dehydrogenase	306 U/L	125–230 U/L
Creatine kinase	45 U/L	29–168 U/L
Troponin I	<2.3 pg/mL	13.8–17.5 pg/mL
Creatine kinase-mb	0.3 ng/mL	0.3–3.4 ng/mL
C-reactive protein	29. 06 mg/dL	< 0.5 mg/dL

**Figure 2:**
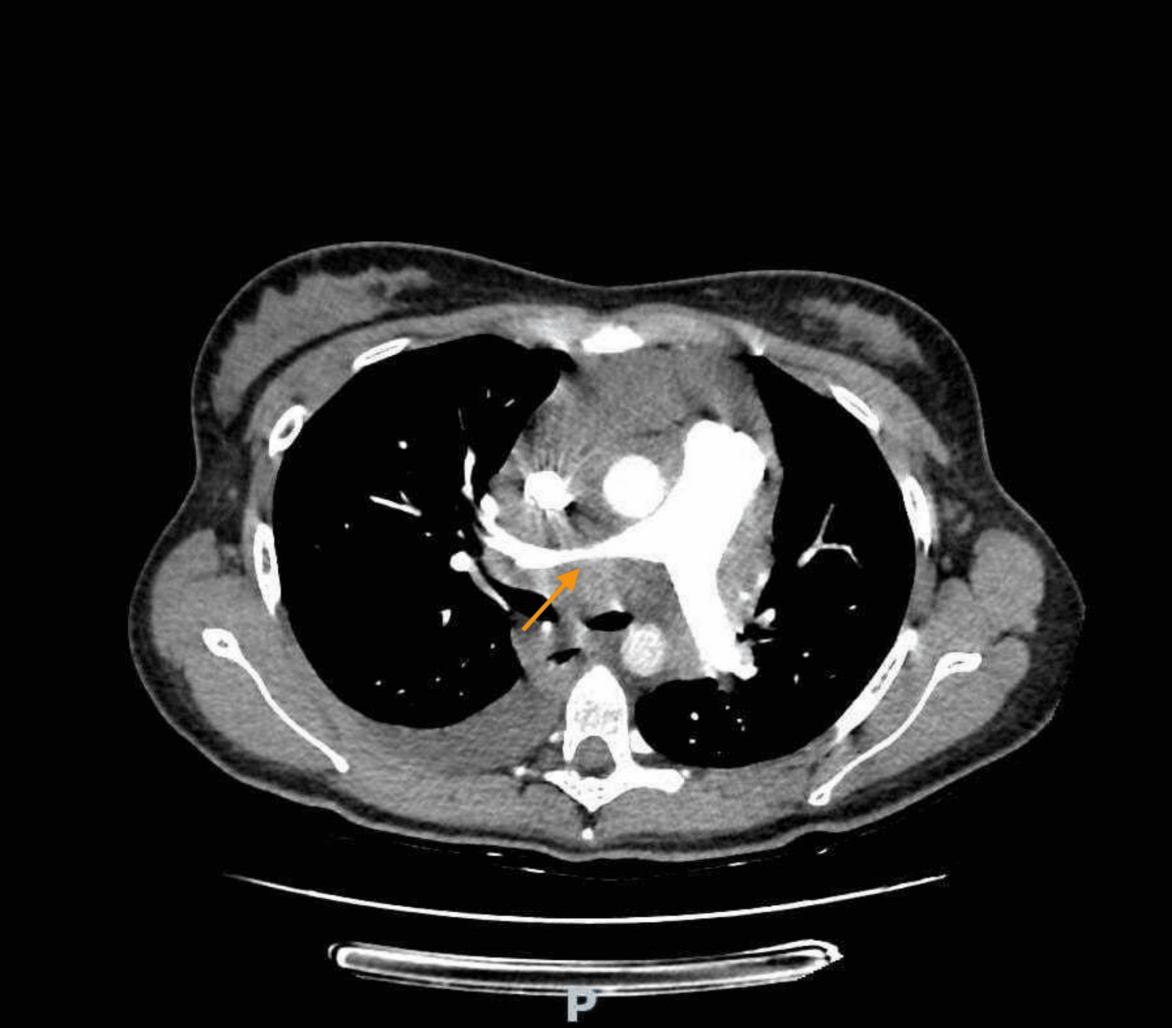
Thoracic CT angiography performed at admission The image shows significant adenopathic densification/mass/conglomerate of the middle/posterior mediastinum, roughly measuring 100x60 mm, involving the main branches of the pulmonary artery, conditioning narrowing of its permeable caliber with 6 mm of caliber of the right main branch of the pulmonary artery (orange arrow). It also involves the central tracheobronchial axis and esophageal path.

**Figure 3:**
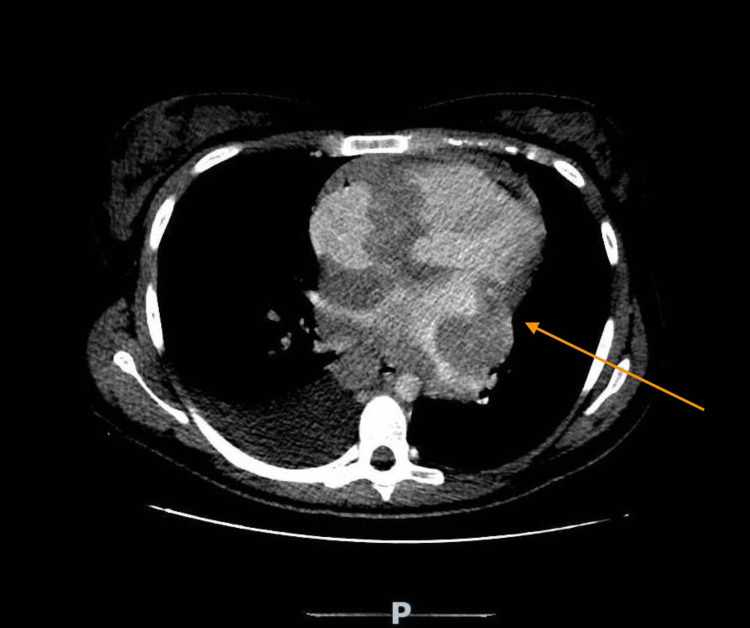
Thoracic tomography The image shows a large mediastinal conglomerate, with adenopathic characteristics, which circumscribes the large vascular structures, especially the pulmonary arteries and veins, also exerting considerable extrinsic compression on the cardiac chambers, in particular the left ventricle (orange arrow), and conditioning significant molding on the coronary sinus and the distal inferior vena cava, which may represent lymphoproliferative disease (Hodgkin's lymphoma). Adenomegaly is isolated in the right cardiophrenic space, measuring 53x23 mm. No axillary or retrocrural adenopathy is seen, nor in other areas of abdominal/pelvic ganglionic drainage.

Aiming to both exclude pulmonary thromboembolism and evaluate the mediastinum enlargement, thoracic CT angiography was performed, revealing a mediastinal mass of 100x60 mm, narrowing the right pulmonary artery and compressing the left ventricle, tracheobronchial axis, and esophageal tract (Figures 2, 3). Due to the suspicion of neoplasia, a thoraco-abdomino-pelvic tomography scan was performed, which did not show other adenopathies or secondary lesions. 

Given the extent of the lesion and the suspicion of lymphoproliferative disease needing urgent diagnosis, it was decided to perform diagnostic mediastinotomy.

However, before admission to the surgery room, the patient experienced a sudden onset of atrial fibrillation with rapid ventricular response associated with severe lactic acidemia, hyperkalemia, and hypocalcemia (Table 3). Treatment with intensive hydration, rasburicase, dexamethasone, and hyperkalemia correction with intravenous insulin and calcium gluconate was immediately initiated. Despite the development of clinical instability, it was still possible to perform the biopsy procedure, and the patient was subsequently admitted to the ICU with mechanical ventilation and vasopressor support.

**Table 3:** Blood gas analysis before surgery

Test	Measured	Reference Value
pH	7.00	7.35–7.45
pCO2	38 mmHg	35–48 mmHg
pO2	106 mmHg	83–108 mmHg
HCO_3_^-^	9.6 mmol/L	21.0–28.0 mmol/L
K^+^	5.4 mmol/L	3.4–4.5 mmol/L
Ca^2+^	0.9 mmol/L	1.15- 1.27 mmol/L
Lactate	12.5 mmol/L	<1.3 mmol/L

On ICU admission hypocalcemia, hyperphosphatemia, and hyperuricemia were evident (Table 4). Despite the absence of prior phosphate or uric acid levels, the emergence of hyperkalemia and hypocalcemia associated with the development of life-threatening arrythmia in a patient suspected of having a lymphoproliferative disease is sufficient to meet criteria for clinical TLS.

**Table 4:** Analysis at admission in the intensive care unit

Test	Results	Reference Value
Hemoglobin	13.1 g/dL	11.5-15 mg/dL
Leucocytes	13,730 uL	4 500–11,400 uL
Platelets	218,000 uL	150,000–350,000 uL
Prothrombin time	16.5 sec	9.4–12.5 sec
International normalized ratio	1.59	0.80–1.20
Activated partial thromboplastin time	25.8 seg	25.1–36.5 seg
Urea	36 mg/dL	15–40 mg/dL
Creatine	0.8 mg/dL	0.6–1.1 mg/dL
Sodium	147 mEq/L	136–146 mEq/L
Potassium	4.2 mEq/L	3.5–5.1 mEq/L
Calcium	7.7 mg/dL	8.8–10.0 mg/dL
Albumin	3.5	3.5–5.0 g/dL
Phosphate	6.7 mg/dL	2.7–4.5 mg/dL
Alanine aminotransferase	359 U/L	< 55 U/L
Aspartate aminotransferase	722 U/L	5–34 U/L
Gamma-glutamyl transferase	43 U/L	9–36 U/L
Lactate dehydrogenase	1426 U/L	125–230 U/L
High-sensitivity troponin I	40.3 pg/mL	13.8–17.5 pg/mL
Uric acid	9.7 mg/dL	3.4–7 mg/dL
C-reactive protein	11. 2 mg/dL	< 0.5 mg/dL

Although the recommended treatment was initiated, clinical deterioration persisted with progression to cardiogenic and obstructive shock requiring multi-organ support, including mechanical ventilation, vasopressor support, and continuous renal replacement therapy.

**Figure 4:**
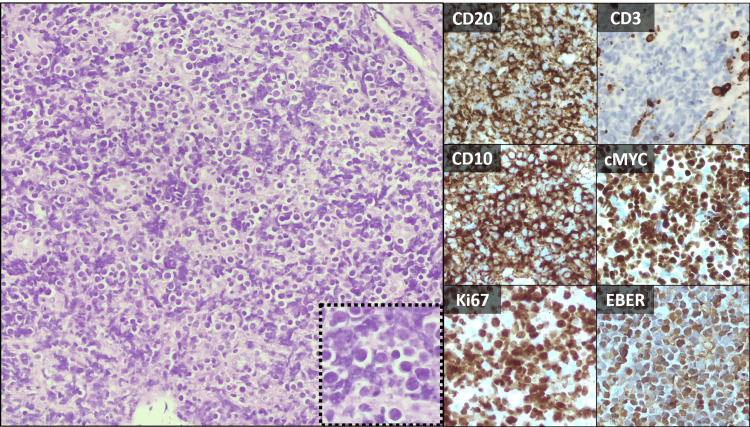
Histological section of the biopsy of mediastinal mass Biopsy showed a proliferation of small-to-medium small monotonous lymphoid cells that were positive for CD20, CD10, cMYC, and EBER, and negative for CD3, BCL2, BCL6, and cyclin D1. The proliferation rate (expression of Ki-67) was near 100%.These aspects were compatible with Burkitt lymphoma.

Biopsy showed aspects compatible with BL with a 100% Ki-67 (Figure 4). 

After hemodynamic stabilization, targeted chemotherapy with hyper-CVAD (cyclophosphamide, vincristine, doxorubicin, and dexamethasone) and rituximab was initiated. After a transient clinical improvement, there was further worsening, followed by bone marrow aplasia and refractory septic shock. Deterioration persisted besides mechanical ventilation, vasopressor support, transfusion support, and continuous renal replacement therapy, as well as empirical antibiotic, antifungal, and antiviral therapy. Although no etiological agent was identified, intestinal mucositis was considered to be the most likely source. The patient died on the 18th day of ICU hospitalization.

## Discussion

In clinical practice, suspecting this diagnosis in a young, previously healthy patient with non-specific symptoms can be challenging. However, persistent respiratory symptoms with multiple healthcare visits are a concerning sign that cannot be overlooked. It should also be noted that hyperlactacidemia without hemodynamic instability or delay in capillary reperfusion time points to lactic acidosis type B, prompting us to suspect less frequent causes, such as neoplasms. Malignant cells can use preferably aerobic glycolysis to support their needs, producing high levels of lactate. This phenomenon, known as the "Warburg effect", is associated with worse prognosis [15]. Also, mediastinal enlargement should not be underappreciated. 

BL presenting as a primary mediastinal tumor is extremely rare, as it typically manifests in the jaw and kidneys in endemic forms or as an intra-abdominal mass in the sporadic variant [1,2]. Most reported cases of thoracic BL in adults are intracardiac. Chan et al. reported 22 intracardiac BL cases [16], with six more added by Schmiester et al. in 2022 [17]. Since then, we have identified three additional cases of cardiac BL [18,19,20]. Most patients were male, aged 4 to 78 years, with symptoms of fatigue and dyspnea. More than 30% of patients died, with 83% within days of diagnosis. Rapid tumor growth in these patients increases the risk of cardiac failure, making prompt initiation of chemotherapy the only life-saving intervention. Cases similar to ours, presenting as an extracardiac mediastinal mass in adults, are extremely rare worldwide. However, the extrinsic compression of great vessels and cardiac chambers posed similar challenges to the cases of cardiac BL leading to hemodynamic instability.

In our patient, instability was precipitated by the onset of spontaneous TLS. Cancer cell lysis not only causes electrolyte imbalances but also releases large amounts of cytokines that cause a systemic inflammatory response syndrome and multiorgan failure [6]. Approximately 30% of cases of TLS occur in NHL [10], particularly BL. However, it was not possible to initiate prophylactic measures because the onset of spontaneous TLS preceded the histological diagnosis.

TLS severity is classified through the Cairo-Bishop grading according to creatinine value and the presence of arrhythmia and seizures [1,8]. In this case, we were confronted with a grade 4 TLS due to the presence of unstable arrhythmia, increasing therapeutic efforts. Although recommended treatment for severe TLS was followed, several other factors contributed to the poor prognosis, including tumor aggressiveness, severe location with large vessel involvement, and left ventricular compression. This scenario precipitated a mixed obstructive and cardiogenic shock with multi-organ support needs, imposing the start of life-saving chemotherapy in an environment of hemodynamic instability and immune vulnerability.

## Conclusions

The reported case underlines the importance of maintaining a high level of suspicion for hematological malignancies in young patients with persistent constitutional symptoms. Blood gas analysis and chest X-ray, simple and highly available diagnostic tools, were essential because lactic acidosis type B and mediastinal enlargement prompt us to suspect other hypotheses besides respiratory infections such as lymphoproliferative disease.

Nonetheless we suspected a high-grade lymphoma, the final diagnosis was still unlikely as BL is atypical in the thoracic location and spontaneous TLS is a rare entity. Therefore, the rapid installation of TLS was unexpected and triggered a scenario of instability that posed significant difficulties to control. In that sense, we highlight the need to be alert for the risk of TLS in patients with suspected high-grade lymphoma even before confirmed diagnosis. New risk stratification tools that can be used before the histological diagnosis can be useful in encouraging earlier prophylactic measures.
